# Effects of Dark Chocolate on Physiological and Anaerobic Performance Among Healthy Female and Male Adults †

**DOI:** 10.3390/nu17213317

**Published:** 2025-10-22

**Authors:** Govindasamy Balasekaran, Yew Cheo Ng, Scott Foong, Xin Rui Rachael Ong, Peggy Boey

**Affiliations:** Physical Education and Sports Science, National Institute of Education, Nanyang Technological University, Singapore 637616, Singapore; nie22.nyc7540@e.ntu.edu.sg (Y.C.N.); kfoong002@e.ntu.edu.sg (S.F.); nie22.roxr@e.ntu.edu.sg (X.R.R.O.); peggy_boey@hotmail.com (P.B.)

**Keywords:** sprint test, milk supplementation, heart rate, rate of perceived exertion, sporting performance, fatigue index

## Abstract

**Background/Objectives**: To investigate the effects of dark chocolate milk on physiological variables such as heart rate (HR), rate of perceived exertion (RPE), fatigue index and power output during an anaerobic sprint test. **Methods**: Twenty healthy participants underwent a randomised single-blinded experimental design and completed two trials—DC and iso-caloric white chocolate (WC) (used as a flavonoid-free control). Participants completed a running anaerobic sprint test (RAST, 35 m × 6 sprints × 2 sets, 4 min rest) with RPE and HR recorded after 2nd, 4th and 6th sprints. **Results**: Descriptive statistics of participants were for males: age: 23.8 ± 1.21 yrs; height: 174.51 ± 5.78 cm; weight: 73.91 ± 9.18 kg; body mass index (BMI): 24.18 ± 2.21 kg·m^−2^; body fat percent (BF%): 19.18 ± 6.17%; lean muscle mass percentage: 77.95 ± 6.16%; females: age: 26.33 ± 4.95 yrs; height: 160.69 ± 5.52 cm; weight: 55.72 ± 7.03 kg; BMI: 21.51 ± 2.02 kg·m^−2^; BF%: 27.24 ± 3.74%; lean muscle mass percentage: 69.20 ± 3.70%. A paired *t*-test revealed significant differences between trials for 2nd RAST average timings (DC 2nd RAST: 6.43 ± 0.97 s vs. WC 2nd RAST: 6.62 ± 1.05 s, *p* = 0.012); 2nd RAST total effort time (DC 2nd RAST: 38.58 ± 5.82 s vs. WC 2nd RAST: 39.72 ± 6.28 s, *p* = 0.012). **Conclusions**: Results indicated that DC supplementation significantly improved anaerobic sprint timings. Athletes, sports practitioners and coaches may consider implementing DC prior to training workouts and competitions to enhance sporting performance.

## 1. Introduction

Elite, competitive athletes and recreational sports enthusiasts commonly seek safe ergogenic tools to assist them in their sporting performances. Minor changes (0.5–1.5%) in sporting performance are considered vital for elite athletes to determine a personal best or medal placing at international meets [1]. Particularly for endurance athletes, they require higher metabolic and nutritional demands [2]. As endurance athletes do face tough conditions during training sessions, they often search for alternative safe dietary solutions to enhance sporting performance, recovery and metabolic health [3]. Especially at elite levels of competitions, these athletes may look for certain supplements that improve sporting performance to provide them an edge over their competitors [4].

Studies have suggested that dark chocolate (DC), another dietary supplement, contains biologically active methylxanthine components (catechins, procyanidins, theobromine, (-)-epicatechin)) found in cocoa that may positively improve the cardiovascular system [5,6,7,8]. The (-)-epicatechin found in DC is converted to 0-methylated that metabolises dominant inhibitors of NADPH oxidase, which suppresses the generation of free radical oxygen (O_2_) and increases bioavailability of NO [9,10]. Moreover, (−)-epicatechin blocks NADPH oxidase that is liable to produce harmful O^2^ molecules or free radicals. Theobromine is a xanthine alkaloid and a compound derived from caffeine metabolism, which is also found at high levels in dark chocolate [11]. Studies found that theobromine indicated efficient absorption into the blood, which may reduce oxidative stress found in the body [12]. This could be beneficial for athletes as high-intensity exercises may produce free radicals that contribute to muscle fatigue and damage [5,6,7,13,14].

Additionally, endurance exercise is typically associated with sustained mitochondrial respiration and electron leakage over time, leading to gradual increases in reactive oxygen species (ROS). In contrast, anaerobic, high-intensity training such as sprinting or repeated maximal bouts relies heavily on glycolysis, which generates rapid ATP turnover, elevated lactate, and increased hydrogen ion (H^+^) accumulation. These metabolic disturbances can trigger acute bursts of oxidative stress and muscle damage. Therefore, the antioxidant and vasodilatory properties of cocoa flavonoids may play a unique role in mitigating the short-term oxidative stress observed during anaerobic performance.

Furthermore, high lactate concentrations accumulated during these intense exercises cause an increase in acidity in muscles (an increase in H^+^ ions) and hence the athlete faces fatigue and muscle soreness that would lead to termination of exercise or slowing down during exercise [15,16]. Fredholm et al. [17] revealed that theobromine provides several physiological benefits that include an increase in HDL cholesterol levels, stimulates the heart, and modulates the lung’s bronchial smooth muscles that is a dominant aspect in the transference of intracellular signals [18].

Moreover, studies have also found that consuming DC with high cocoa content (70% or more) may improve endurance sporting performance. This could be due to the vasodilatory effects of flavonoids that could improve blood flow and O_2_ delivery to the working muscles to improve athletic performance and vascular function and lower blood pressure [11,19]. Nemoto et al. [20] found that DC reduced fatigue among individuals that could lead to an enhanced executive function. The experimental group consumed five pieces of DC whereas the control group was told to refrain from consuming chocolate during the intervention study. Results found that there was a significant reduction in physical and mental fatigue between both groups (*p* = 0.035). As mentioned earlier, DC contains flavonoids and antioxidants that are associated with increased vascular function and enhanced blood flow [21,22]. With better blood circulation, the flavonoids may enhance the delivery of O_2_ to muscles during exercise and delay the onset of fatigue.

To date, to the authors’ knowledge, no studies have investigated the impact of dark chocolate on anaerobic performance. Therefore, this study investigated whether dark chocolate could influence the metabolic processes involved in ATP resynthesis, which underpins repeated high-intensity muscular contractions, and in turn enhance sprint performance. This study may provide further insights in understanding the challenges for athletes and sports enthusiasts that seek to optimise anaerobic performance through natural dietary means.

## 2. Materials and Methods

**Figure 1:**
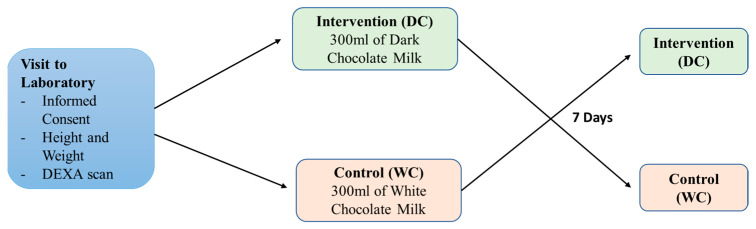
Experimental Design and Protocol.

Twenty healthy participants underwent a randomised single-blinded experimental design and completed 2 sessions. The inclusion criteria required participants to be 21–35 years old, have no history of musculoskeletal injuries in the past 6 months, not be on any medication, perform at least 150 min of moderate-intensity exercise or 75 min of vigorous-intensity exercise per week, and exercise 3 times weekly. Permuted block randomisation was used to randomly assign the first half of the participants to the DC group and the other half were assigned to the WC group. They had a 7-day wash-out period before they completed the other trial (DC or WC) (Figure 1). Participants were informed to keep their physical activities and diet similar throughout the entire study duration. Female participants were told to perform their first trial during the follicular phase (7 days) of their menstrual cycle and the second trial after the 7-day wash-out period. All trial sessions were conducted in the morning from 900 h to 1200 h as participants were required to comply with an overnight fast. As the primary objective was to measure the acute effects of DC milk consumption on anaerobic sprint performance and fatigue under conditions that closely reflect real-world scenarios, the design mimicked everyday life. This is where dietary habits vary, and complete elimination of polyphenol intake may be impractical outside of controlled inpatient studies. The experimental protocols took place in the Human Bioenergetics Laboratory (HBL) basketball and handball courts at Physical Education & Sports Science (PESS), National Institute of Education (NIE), Nanyang Technological University (NTU).

During the first test session, participants were briefed on an overview of the study, its objective, experimental protocol, and potential risks/side effects. They were required to provide their consent via an informed consent form, and they completed a Physical Activity Readiness Questionnaire for Everyone (PAR-Q+) for any contraindications to exercise testing prior to commencement of the test protocol.

Prior to the test, participants’ height and weight were measured using the InBody 770 machine (Hessenbergweg 75A 1101CX, Amsterdam, The Netherlands). Then, their body compositions were analysed using a dual-energy X-ray absorptiometry (DEXA) machine (Lunar Prodigy, General Electric Healthcare, Chicago, IL, USA), which was operated by a certified laboratory technician of the university. Prior to the DEXA scan, participants were required to complete a DEXA consent form. If they ticked “Yes” to any questions, they were not allowed to proceed with the scan. Participants’ lean muscle mass, bone mineral density (BMD), and bone mineral content (BMC) were measured during the scan. Their HR was measured using a polar heart rate sensor (Polar RS400, Kempele, Finland) connected to an application on a mobile phone (Polar Beat). A familiarisation session was conducted where the participant was anchored and trained to use the OMNI RPE (RPE) pictorial scale [23].

DC and WC groups consumed 300 mL of DC milk and 300 mL of milk mixed with 5–7 WC flavour drops, respectively. The DC milk contains approximately 51.48 g/30.64% cocoa powder in 300 mL of milk. Products that are made from cacao beans or made into cocoa powder contain theobromine, catechins (natural antioxidants found in cacao beans), and procyanidins. Sucrose, also known as table sugar, is found in dark chocolate. According to the nutrient label, it is indicated that DC contains 8.7 g of total sugar and 1.3 g of total fat; whereas WC contains 4.3 g of total sugar and 3.8 g of total fat. The milk was provided in an opaque covered cup and the participants drank it through an opaque metal straw to ensure that the testing was fair and unbiased. They were given 1.5 h for digestion and rested during this period [24]. Participants put on the polar HR monitor to monitor their HR during the test. Ten minutes before the start of the test, participants carried out a set of standardised warmups that consisted of static stretching exercises for major muscle groups for the lower body. Static stretching of major muscle groups was carried out to ensure that the warmup was sufficient for the participants’ muscles to be activated and it would not negatively affect their sprint performance. Their resting HR and RPE were recorded prior to the start of the test.

The Running-Based Anaerobic Sprint Test (RAST) was employed and participants performed six 35 m sprints with 10 s of passive recovery between each sprint. Timing gates (Swift Performance, SpeedLight, Brisbane, QLD, Australia) was used to measure the time taken for each 35 m sprint and they were placed at the beginning and end of the 35 m sprint. Another test administrator used a stopwatch to time the 10 s rest between each sprint [25]. When the test administrator said “Go”, the participants were told to sprint quickly to the opposite gate. During the 10 s passive recovery, the test administrator gave a countdown of “5–4–3–2–1” and on “Go” the participant would sprint back to the start. This process continued until all 6 sprints were completed. Two test administrators were present during all tests where one was responsible for monitoring the timing gate system and the other kept track of the rest period and provided participants verbal encouragement [26].

Participants’ RPE and HR were recorded after the 2nd, 4th, and 6th sprint of each RAST. After the first set was completed, they rested for 4 min [27] and continued to the 2nd RAST. The results of the tests were calculated using the following Formulas (1) and (2) [28].

Power = (Body Mass × Distance^2^)/Time^3^, (1)

Fatigue Index = [(Maximum Power − Minimum power)] × 100,(2)

Using a power of 0.95 at an alpha level of 0.05 calculated by G*Power (Version 3.1.9.6), 20 participants were required in order to detect a large effect size (d = 1) and carry out interactive statistical analysis [29,30]. All statistical analyses were performed using the Statistical Package for Social Science (IBM SPSS 23.0, Chicago, IL, USA) software. A paired *t*-test was used to compare physiological and performance data between the 1st and 2nd RASTs and FI. Two-way repeated measures ANOVA was used to calculate the average sprint time, average power output, RPE, and HR for each sprint set and trial and between genders. Pearson’s correlation was conducted between weight, body fat, lean mass, BMD, DC total RAST, WC total RAST, relative power DC 2nd RAST, and relative power WC 2nd RAST. Significant values were set at (*p* < 0.05).

Descriptive statistics of participants such as age, height, weight, BMI, BF%, lean mass, and fat mass were calculated as means (M) ± standard deviations (SD) (Table 1).

## 3. Results

### 3.1. Physiological Measures

#### 3.1.1. Descriptive Statistics of Participants

The descriptive statistics of twenty healthy adults (males: age: 23.8 ± 1.21 yrs; height: 174.51 ± 5.78 cm; weight: 73.91 ± 9.18 kg; body mass index (BMI): 24.18 ± 2.21 kg·m^−2^; body fat percent (BF%): 19.18 ± 6.17%; lean muscle mass percentage: 77.95 ± 6.16%; females: age: 26.33 ± 4.95 yrs; height: 160.69 ± 5.52 cm; weight: 55.72 ± 7.03 kg; BMI: 21.51 ± 2.02 kg·m^−2^; BF%: 27.24 ± 3.74%; lean muscle mass percentage: 69.20 ± 3.70%) are shown in Table 1.

#### 3.1.2. Total Effort Time, Fatigue Index (FI%), and Mean Power Between Trials

**Figure 2:**
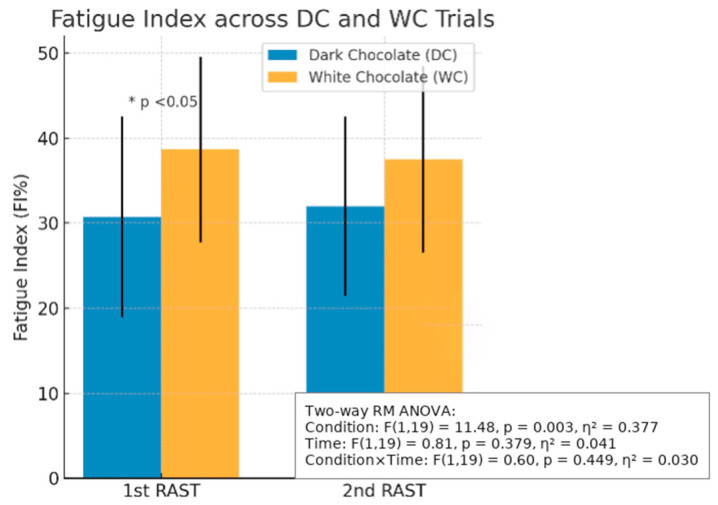
Fatigue Index (FI%) between Dark Chocolate (DC) and White Chocolate (WC) across two RAST trials. Values are mean ± SD. * indicates *p* < 0.05 for pairwise comparison between conditions in the 1st RAST. Two-way repeated measures ANOVA results: main effect of Condition, F(1,19) = 11.48, *p* = 0.003, ηp^2^ = 0.377; no main effect of Time, F(1,19) = 0.81, *p* = 0.379, ηp^2^ = 0.041; and no Condition × Time interaction, F(1,19) = 0.60, *p* = 0.449, ηp^2^ = 0.030.

Results from the Shapiro–Wilk test indicated that the data were normally distributed for total effort time, FI%, and mean power (*p* > 0.05). Statistical paired *t*-test data analysis revealed significant differences between DC and WC trials for 1st RAST FI% (DC: 30.71 ± 11.82% vs. WC: 38.67 ± 10.91%, *p* = 0.006) (Table 2, Figure 2). There were also significant differences between trials for 2nd RAST total effort time (DC: 38.58 ± 5.82 s vs. WC: 39.72 ± 6.28 s, *p* = 0.012); 2nd RAST average time (DC: 6.43 ± 0.97 s vs. WC: 6.62 ± 1.05 s, *p* = 0.012); mean power (DC: 354.09 ± 173.88 W vs. WC: 323.81 ± 149.32 W, *p* = 0.009); relative mean power (DC: 5.24 ± 1.99 W vs. WC: 4.81 ± 1.70 W, *p* = 0.007) (Table 3). There were significant differences between trials for males’ FI% in the 1st RAST (DC: 32.47 ± 8.93% vs. WC: 39.60 ± 9.14%, *p* = 0.020); for females’ resting HR in the 1st RAST (DC: 87.20 ± 16.26 beats·min^−1^ vs. 96.70 ± 18.85 beats·min^−1^, *p* = 0.042) (Table 4). Significant differences were found between trials for males’ mean power in the 2nd RAST (DC: 509.80 ± 69.36 W vs. WC: 454.44 ± 63.37 W, *p* = 0.010); males’ relative mean power in the 2nd RAST (DC: 6.93 ± 0.76 W·kg^−1^ vs. 6.17 ± 0.74 W·kg^−1^, *p* = 0.009) (Table 5).

#### 3.1.3. Average Sprint Time Between Sets, Trials, and Gender

**Figure 3:**
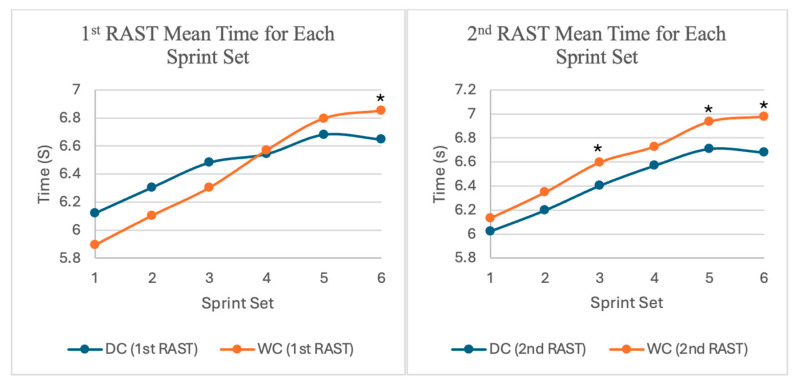
Participants’ mean time for each sprint set for 1st and 2nd RAST for both DC and WC trials. * indicates significant difference between sprint set *p* < 0.05.

Mauchly’s test of sphericity was conducted and results indicated that the assumption of sphericity was violated (*p* < 0.05), therefore the Greenhouse–Geisser correction was applied (ε=0.72) to account for violation of sphericity and to ensure that the results are statistically valid. Two-way repeated measures ANOVA Greenhouse–Geisser results showed that there was a significant effect between average sprint time for each sprint set and trial (F(2.55, 96.91) = 5.19, *p* = 0.004, ηp^2^ = 0.12) for the 1st RAST and 2nd RAST in DC and WC trials. There were also significant differences between 1st RAST DC and WC 6th sets (1st RAST DC 6th set: 6.65 ± 0.90 s vs. 1st RAST WC 6th set: 6.85 ± 0.89 s, *p* = 0.007); 2nd RAST DC and WC 3rd, 5th, and 6th set (2nd RAST DC 3rd set: 6.40 ± 0.97 s vs. 2nd RAST WC 3rd set: 6.60 ± 1.04 s, *p* = 0.005); 2nd RAST DC 5th set (6.71 ± 1.02 s) vs. 2nd RAST WC 5th set (6.94 ± 1.16 s, *p* = 0.015); 2nd RAST DC 6th set (6.68 ± 0.90 s) vs. 2nd RAST WC 6th set (6.98 ± 1.05 s, *p* = 0.005) (Figure 3). Two-way repeated measures ANOVA results also showed that there was a significant effect between average sprint times for each sprint set and trial for females (F(1.94, 34.82) = 4.11, *p* = 0.026, ηp^2^ = 0.19) for 1st RAST DC and WC trials. However, there was no significant effect between average sprint times for each sprint set and trial for males (F(5, 90) = 1.56, *p* = 0.180, ηp^2^ = 0.08) for 1st RAST DC and WC trials or between average sprint times for each sprint set and trial in 2nd RAST DC and WC trials for both males (F(2.21, 39.72) = 0.37, *p* = 0.713, ηp^2^ = 0.02) and females (F(1.56, 28.12) = 1.01, *p* = 0.359, ηp^2^ = 0.05).

#### 3.1.4. Average Power Output Between Trials

Results from the Shapiro–Wilk test indicated that the data were normally distributed for average power output (W(20) = 0.934, *p* = 0.188). There were also no significant effects for average power output between each sprint set and trial in the 1st RAST (F(2.13, 80.81) = 1.91, *p* = 0.152, ηp^2^ = 0.05) or 2nd RAST (F(1.60, 60.84) = 0.14, *p* = 0.826, ηp^2^ = 0.004) in DC and WC trials; between average power output for each sprint set and trial in 1st RAST for both males (F(3.11, 55.96) = 1.19, *p* = 0.321, ηp^2^ = 0.06) and females (F(2.14, 38.45) = 3.24, *p* = 0.047, ηp^2^ = 0.004); or the 2nd RAST in both males (F(2.07, 37.29) = 0.44, *p* = 0.654, ηp^2^ = 0.002) and females (F(1.51, 27.12) = 0.73, *p* = 0.456, ηp^2^ = 0.004) in DC and WC trials. However, there was a significant difference between trials for FI% the 1st RAST (DC 1st RAST: 30.71 ± 11.82% vs. WC 1st RAST: 38.67 ± 10.91%, *p* = 0.006). No significant difference was found between trials for FI% in the 2nd RAST (DC 2nd RAST: 30.50 ± 11.82% vs. WC 2nd RAST: 35.47 ± 13.85%, *p* = 0.099) (Figure 2).

#### 3.1.5. Average Heart Rate and Rate of Perceived Exertion Between Trials

Results indicated that the data for average HR and RPE were normally distributed (W(20) = 0.947, *p* = 0.331 and W(20) = 0.958, *p* = 0.509, respectively) using the Shapiro–Wilk test. Two-way repeated measures ANOVA results showed that there were no significant effects between average HRs for each sprint set (rest, 2nd, 4th, and 6th) and trial in the 1st RAST (F(1.86, 70.84) = 0.53, *p* = 0.581, ηp^2^ = 0.01) or 2nd RAST (F(1.46, 55.37) = 0.39, *p* = 0.616, ηp^2^ = 0.01) in DC and WC trials. There were also no significant effects between average HRs for each sprint set (rest, 2nd, 4th, and 6th) and trial in the 1st RAST for males (F(1.79, 32.28) = 0.05, *p* = 0.941, ηp^2^ = 0.003) and females (F(1.46, 26.36) = 1.01, *p* = 0.356, ηp^2^ = 0.05) or 2nd RAST for males (F(1.88, 33.83) = 0.61, *p* = 0.540, ηp^2^ = 0.03) and females (F(1.18, 21.19) = 0.36, *p* = 0.588, ηp^2^ = 0.02) in DC and WC trials. In addition, there were no significant effects between average RPEs for each sprint set (rest, 2nd, 4th, and 6th) and trial in the 1st RAST (F(1.64, 62.28) = 1.07, *p* = 0.338, ηp^2^ = 0.03) or 2nd RAST (F(1.99, 75.43) = 0.65, *p* = 0.524, ηp^2^ = 0.02) in DC and WC trials. Results also showed that there were no significant effects between average RPEs for each sprint set (rest, 2nd, 4th, and 6th) and trial in the 1st RAST for males (F(1.47, 26.40) = 2.49, *p* = 0.115, ηp^2^ = 0.12) and females (F(1.59, 28.60) = 0.22, *p* = 0.753, ηp^2^ = 0.01) or 2nd RAST for males (F(1.99, 35.82) = 1.21, *p* = 0.311, ηp^2^ = 0.06) and females (F(1.92, 34.64) = 0.38, *p* = 0.677, ηp^2^ = 0.02) in DC and WC trials.

## 4. Discussion

The RAST involves the anaerobic glycolysis system, which is the breakdown of carbohydrates to lactic acid without the presence of O_2_ [15]. Furthermore, the rate-limiting enzymes (PFK and PK) that control and limit overall performance of glycolysis and this glycolysis process may be impeded if the rate-limiting steps are processed slowly [15,30]. The main ingredients found in DC (theobromine, catechins, procyanidins, and sucrose) would have reciprocal effects on endothelial function and cardiovascular biomarkers [5,6,7]. As these components are absorbed into the blood from the gut lumen, the metabolites affect the processes in the endothelium [11]. Therefore, these effects optimise O_2_ levels and positively affect PFK and PK to boost the glycolysis system and enhance athletic performance.

Overall results supported the hypothesis of this study where DC significantly improved 2nd RAST total effort time (DC: 38.58 ± 5.82 s vs. WC: 39.72 ± 6.28 s, *p* = 0.012) and average sprint time (DC: 6.43 ± 0.97 s vs. WC: 6.62 ± 1.05 s, *p* = 0.012) (Table 3). This could be due to the high levels of flavonoids that DC contains that increase the amount of NO being released from the endothelium. This would lead to a boost in vasodilation that increases O_2_ delivery to the working muscles and elevating stroke volume. Moreover, the sucrose found in DC milk comprises glucose and fructose that enters glycolysis quickly, thus contributing to the production of ATP. Therefore, sucrose provides immediate ATP for glycolysis while the flavonoids support long-term ATP production through improved blood flow. This synergistic effect of sucrose and flavonoids seem to have a positive role in performance. However, other studies have also found that a higher ratio of flavonoids to sucrose (found in dark chocolate) appeared to be more effective for sporting performance. Previous research has suggested that cocoa flavonoids may increase signaling pathways like peroxisome proliferator-activated receptor-gamma coactivator 1-alpha (PGC-1α), which is a master regulator of mitochondrial biogenesis and enhances NO bioavailability, leading to improved vasodilation and oxygen delivery. Furthermore, it interacts with the electron transport chain (ETC) that promotes more efficient ATP production. While these mechanisms provide plausible explanations for potential long-term adaptations, it is essential to note that such molecular responses were not directly measured in this study.

In this study, WC also contained sucrose (4.3 g) but sucrose may only have played a minor role in the significant improvement in 2nd RAST total effort time (Table 3). Therefore, flavonoids increase mitochondrial number and enhance energy production efficiency, improving cellular metabolism. Since there were no significant differences in average power output and average sprint time for each sprint set and trial, participants may have already been performing near their maximal potential for both trials as there were also no significant differences between trials in terms of HR and RPE. Therefore, the ability of either beverage to further enhance performance could be negligible.

Although there was no significant difference between average power output, there was a significant difference in FI% between trials for the 1st RAST (DC: 30.71 ± 11.82%, vs. WC: 38.67 ± 10.91%, *p* = 0.006) (Table 2, Figure 2) due to the increase in O_2_ delivery to the working muscles, thus reducing the fatigue by delaying the onset of anaerobic metabolism. Additionally, the antioxidants found in DC offset the reactive O_2_ species that reduce oxidative stress and protect muscle cells [12]. In anaerobic sports, a lower FI% enables athletes to maintain peak performance over time. The antioxidant properties of flavonoids prevent oxidative damage and delay fatigue. The sucrose synergises with flavonoids to enhance glycogen replenishment and utilisation during exercise. Moreover, milk proteins slow digestion and sustain the release of amino acids that allows gradual replenishment of glycogen. Therefore, there was a positive impact on cardiovascular function and blood flow and an increment in cardiac output, allowing the individual to maintain the power output for a longer duration and he/she was able to improve in his/her sprint timing.

The improvement in FI and sprint performance may be explained by acute metabolic benefits of DC, including enhanced blood flow, antioxidant effects, and rapid glycolytic energy supply from sucrose. However, RPE and HR did not differ significantly between conditions as these measures reflect broader perceptions of effort or systemic cardiovascular strain, which are less sensitive to subtle metabolic and muscular changes reflected by FI and sprint performance outcomes. As resting HR was measured prior to participants’ warmup, their resting HR values may appear slightly higher as compared to resting HR taken immediately after waking up from sleep. Interestingly, the results indicated a significant difference for resting HR between trials, which further supports the notion that DC can positively modulate autonomic function, therefore decreasing resting HR. Improved parasympathetic reactivation allows athletes to recover more quickly after high-intensity efforts. A faster heart rate recovery (HRR) between intervals is associated with better endurance and aerobic fitness. Enhanced autonomic balance, particularly parasympathetic activity, can increase stroke volume, leading to more efficient oxygen and nutrient delivery to working muscles. Better autonomic regulation ensures appropriate vasodilation and blood redistribution, delivering oxygen and nutrients to active muscles while maintaining overall systemic balance. The compounds found in DC, particularly (-)-epicatechin, promote arterial vasodilation, enhance blood flow, and improve endothelial function. There were no significant differences between average HR and RPE in the 6th set of both DC and WC trials (Table 2 and Table 3), with significant correlation between DC and WC HR and RPE in the 4th and 6th sets (4th set: r = 0.512, *p* = 0.021; 6th set: r = 0.588, *p* = 0.006). Correlation results also indicated several significant correlations between overall HR and RPE for both trials. It can be concluded that participants reached similar maximal effort for both trials. This study’s correlation results coincide with other studies that indicated positive linear regressions and significant correlations between HR and RPE [15,20,31,32,33].

This study also found significant correlation between relative mean power output DC 2nd RAST and DC total RAST timings (relative mean power output DC 2nd RAST: 6.93 ± 0.76 W·kg^−1^ vs. DC total RAST timings: 77.37 ± 11.63 s, r = −0.929, *p* = 0.000) and relative mean power output WC 2nd RAST and WC total RAST timings (relative mean power output WC 2nd RAST: 6.17 ± 0.74 W·kg^−1^ vs. WC total RAST timings: 78.24 ± 10.72 s, r = −0.955, *p* = 0.000). These results indicated that the higher the relative mean power output, the faster the RAST timings. This also further supports previous studies that have investigated the correlation between power output and speed. Furthermore, the correlation results also revealed that the lower the body fat, the faster the RAST timings. This also coincides with another study that found significant negative correlation between countermovement jump, squat jump, and body fat percentage [34,35,36].

However, results also indicated significant gender differences regarding height, weight, BMI, BF%, lean muscle mass, BMD, and BMC (Table 1). This may reveal that DC’s effects may differ between individuals and genders due to the differences in metabolism and hormonal response. The higher fat mass in females and greater lean mass in males can alter how nutrients are absorbed, distributed, and metabolised and that may have influenced FI% during the anaerobic sprint. Furthermore, athletes should continue to prioritise a balanced diet to support healthy body composition, as excessive intake of any food, including dark chocolate, may contribute to weight gain and other adverse health outcomes [37,38]. Some athletes may experience no effects due to such differences in metabolism or flavonoid absorption, which may explain the lack of a significant difference in average power output between trials. Moreover, this may suggest that DC may not directly translate to changes in power output. Perhaps flavonoids could further reduce oxidative stress and improve recovery and that could enhance power output over a longer period; results may vary depending on each individual [39,40].

## 5. Limitations

Participants’ diet could have been better controlled as there were no strict dietary restrictions. Participants’ intake of polyphenols could have also been better controlled as it may have had some influence on the findings. However, this was due to the primary assessment of DC consumption on anaerobic sprint, fatigue, and power output under conditions reflecting real-world scenarios. Athletes and/or recreational individuals may not follow strict elimination diets prior to performance. Thus, this design was developed to mimic everyday life where dietary habits vary, and complete elimination of polyphenol intake may be impractical outside of controlled inpatient studies. Future studies could also investigate the long-term and short-term effects of DC as it may require a longer duration and regular consumption to induce significant changes for more physiological measures. Additionally, cardiovascular biomarkers could also be included to provide more direct evidence of these physiological mechanisms to strengthen the link between NO production, delayed fatigue, and performance improvement. Future studies could also investigate the effects of DC on plasma blood pre- and post-exercise to ascertain the significance of this study’s results.

## 6. Conclusions

The results from this study revealed that DC significantly improves anaerobic sprint timings as compared to WC. Although not directly measured, this study could conclude that flavonoids found in DC promoted NO release from the endothelium and thus increased O_2_ delivery to the working muscles during exercise. This allowed the individual to optimise O_2_ levels to prolong and maintain their sprint timings, especially during the 2nd RAST. With this optimisation, rate-limiting enzymes, PFK and PK, are able to work more efficiently during the glycolysis process that increases ATP production to allow a higher stroke volume, work rate, and cardiac output to improve athletic performance. While the absolute differences observed were relatively small, such effects may hold limited clinical significance for the general exercising population. Nevertheless, these marginal differences (0.5–1.5%) may influence competitive outcomes, making the difference between winning and losing.

Furthermore, DC is also easily available and inexpensive to purchase from convenient supermarkets as compared to other types of artificial supplements that are expensive and could contribute to significant side effects to the individual. This study could provide athletes and sports enthusiasts with a safe, natural, and easily accessible dietary option and evidence-based nutritional strategy to enhance anaerobic performance. It could also support sports nutritionists in developing personalised dietary recommendations for athletes (based on athlete’s age, fitness level, and dietary preferences) to utilise DC as a viable ergogenic aid for athletes and sports enthusiasts to engage in high-intensity sporting activities.

On the contrary, athletes and recreational individuals should also be reminded that there are also other factors affecting athletic performance and that their individual regular training is also essential towards enhancing their sporting performance. A balanced diet is still necessary for athletes to maintain a healthy body composition, whereby excessive consumption may lead to unwanted weight gain and other health issues. The beneficial effects of DC on oxidative stress may vary depending on the individual such as genetics, overall diet, and lifestyle. Elite athletes, sub-elite athletes, sports enthusiasts, sports practitioners, and coaches may utilise DC as a safe dietary supplement as a pre-workout drink prior to training sessions or competitions to provide them an advantageous competitive edge.

## Figures and Tables

**Table 1:** Descriptive Characteristics of Participants (n = 20).

Variables	Total Cohort (n = 20) (Mean ± SD)	Males (n = 10) (Mean ± SD)	Females (n = 10) (Mean ± SD)	*p* Value
Age (years)	25.07 ± 3.74	23.8 ± 1.21	26.33 ± 4.95	0.134
Height (cm)	167.60 ± 8.98	174.51 ± 5.78	160.69 ± 5.52	0.000 *
Weight (kg)	64.82 ± 12.26	73.91 ± 9.18	55.72 ± 7.03	0.000 *
BMI (kg·m^−2^)	22.85 ± 2.48	24.18 ± 2.21	21.51 ± 2.02	0.011 *
BF% (%)	23.21 ± 6.46	19.18 ± 6.17	27.24 ± 3.74	0.002 *
Lean Muscle Mass (g)	48,460.16 ± 13,409.05	59,445.22 ± 10,705.85	38,573.60 ± 5333.75	0.000 *
Lean Muscle Mass (%)	73.58 ± 6.68	77.95 ± 6.16	69.20 ± 3.70	0.001 *
BMD (g·cm^2^)	1.23 ± 0.08	1.27 ± 0.08	1.19 ± 0.07	0.027 *
BMC (g)	2888.95 ± 601.67	3304.60 ± 535.09	2473.30 ± 306.60	0.000 *

Values are in mean (M) ± standard deviation (SD). Age (years); height (centimeters, cm); weight (kilograms, kg); body mass index (BMI, kg·m^−2^); body fat percent (BF, %); lean muscle mass (grams, g); lean muscle mass (percentage, %); bone mass density (BMD, grams per unit area, g·cm^2^); bone mineral content (BMC, grams, g). * indicates significant differences between males and females (*p* < 0.05).

**Table 2:** Results for Total Cohort 1st RAST between DC and WC Trials.

	DC	WC	*p* Value
	(Mean ± SD)	(Mean ± SD)	
Total Effort Time (s)	38.78 ± 5.86	38.52 ± 4.62	0.592
Average Time (s)	6.46 ± 0.98	6.42 ± 0.77	0.592
Peak Power (W)	432.04 ± 216.24	440.03 ± 196.63	0.615
Relative Power (W·kg^−1^)	6.38 ± 2.47	6.59 ± 2.27	0.373
Mean Power (W)	345.93 ± 166.96	341.86 ± 147.22	0.754
Relative Mean Power (W·kg^−1^)	5.13 ± 1.89	5.11 ± 1.63	0.907
FI (%)	30.71 ± 11.82	38.67 ± 10.91	0.006 *
Resting HR (beats·min^−1^)	89.40 ± 13.30	95.30 ± 14.95	0.019 *
Mean HR (beats·min^−1^)	157.77 ± 8.24	159.70 ± 8.66	0.236
HR 6th Set (beats·min^−1^)	168.75 ± 7.23	170.15 ± 9.18	0.250
RPE 6th Set	7.85 ± 1.31	7.90 ± 1.29	0.841

Values are means (M) ± standard deviation (SD). DC (dark chocolate); WC (white chocolate); Total Effort Time (seconds, s); Average Time (seconds, s); Peak Power (watts, W); Relative Power (watts per kilogram, W·kg^−1^); Mean power (watts, W); Relative mean power (watts per kilogram, W·kg^−1^); Fatigue Index (FI, percentage, %); resting HR (resting heart rate); mean HR (heart rate, beats per minute, beats·min^−1^); HR 6th set (heart rate, beats per minute, beats·min^−1^); RPE 6th set (rate of perceived exertion). * indicates significant difference between trials for 1st RAST (*p* < 0.05).

**Table 3:** Results for Total Cohort 2nd RAST between DC and WC Trials.

	DC	WC	*p* Value
	(Mean ± SD)	(Mean ± SD)	
Total Effort Time (s)	38.58 ± 5.82	39.72 ± 6.28	0.012 *
Average Time (s)	6.43 ± 0.97	6.62 ± 1.05	0.012 *
Peak Power (W)	443.76 ± 234.33	421.08 ± 209.56	0.191
Relative Power (W·kg^−1^)	6.53 ± 2.71	6.24 ± 2.44	0.207
Mean Power (W)	354.09 ± 173.88	323.81 ± 149.32	0.009 *
Relative Mean Power (W·kg^−1^)	5.24 ± 1.99	4.81 ± 1.70	0.007 *
FI (%)	30.50 ± 11.82	35.47 ± 13.85	0.099
Resting HR (beats·min^−1^)	114.50 ± 16.65	114.35 ± 19.44	0.951
Mean HR (beats·min^−1^)	167.32 ± 7.97	165.50 ± 8.23	0.321
HR 6th Set (beats·min^−1^)	176.60 ± 7.72	173.50 ± 7.92	0.048
RPE 6th Set	8.90 ± 0.97	8.90 ± 1.17	1.000

Values are means (M) ± standard deviation (SD). DC (dark chocolate); WC (white chocolate); Total Effort Time (seconds, s); Average Time (seconds, s); Peak Power (watts, W); Relative Power (watts per kilogram, W·kg^−1^); Mean power (watts, W); Relative mean power (watts per kilogram, W·kg^−1^); Fatigue Index (FI, percentage, %); resting HR (resting heart rate); mean HR (heart rate, beats per minute, beats·min^−1^); HR 6th set (heart rate, beats per minute, beats·min^−1^); RPE 6th set (rate of perceived exertion). * indicates significant difference between trials for 2nd RAST (*p* < 0.05).

**Table 4:** Comparison between Genders for 1st RAST DC and WC Trials.

	Male		*p* Value	Female		*p* Value
	(Mean ± SD)			(Mean ± SD)		
	DC	WC		DC	WC	
Total Effort Time (s)	34.35 ± 1.50	34.89 ± 1.17	0.307	43.21 ± 5.16	42.15 ± 3.78	0.201
Average Time (s)	5.73 ± 0.25	5.82 ± 0.20	0.307	7.20 ± 0.86	7.03 ± 0.63	0.201
Peak Power (W)	627.34 ± 82.55	619.49 ± 67.03	0.785	236.75 ± 84.54	260.57 ± 74.59	0.120
Relative Power (W·kg^−1^)	8.52 ± 0.76	8.47 ± 1.10	0.896	4.25 ± 1.49	4.71 ± 1.34	0.111
Mean Power (W)	492.84 ± 78.80	472.44 ± 56.51	0.408	199.03 ± 68.42	211.28 ± 68.32	0.205
Relative Mean Power (W·kg^−1^)	6.69 ± 0.87	6.42 ± 0.68	0.386	3.56 ± 1.15	3.79 ± 1.14	0.199
FI (%)	32.47 ± 8.93	39.60 ± 9.14	0.020 *	28.94 ± 10.50	37.74 ± 12.88	0.089
Resting HR (beats·min^−1^)	91.60 ± 9.91	93.90 ± 10.60	0.235	87.20 ± 16.26	96.70 ± 18.85	0.042 *
Mean HR (beats·min^−1^)	157.40 ± 10.52	159.87 ± 9.61	0.373	158.13 ± 5.68	159.53 ± 8.12	0.477
HR 6th Set (beats·min^−1^)	170.60 ± 9.14	172.50 ± 10.35	0.353	166.90 ± 4.38	167.80 ± 7.66	0.546
RPE 6th Set	7.80 ± 1.40	8.40 ± 0.97	0.081	7.90 ± 1.29	7.40 ± 1.43	0.138

Values are means (M) ± standard deviation (SD). DC (dark chocolate); WC (white chocolate); Total Effort Time (seconds, s); Average Time (seconds, s); Peak Power (watts, W); Relative Power (watts per kilogram, W·kg^−1^); Mean power (watts, W); Relative mean power (watts per kilogram, W·kg^−1^); FI (Fatigue Index, percentage, %); resting HR (resting heart rate); mean HR (heart rate, beats per minute, beats·min^−1^); HR 6th set (heart rate, beats per minute, beats·min^−1^); RPE 6th set (rate of perceived exertion). * indicates significant difference between trials for gender in 1st RAST (*p* < 0.05).

**Table 5:** Comparison between Genders for 2nd RAST DC and WC Trials.

	Male		*p* Value	Female		*p* Value
	(Mean ± SD)			(Mean ± SD)		
	DC	WC		DC	WC	
Total Effort Time (s)	33.99 ± 1.38	35.41 ± 1.50	0.004 *	43.18 ± 4.76	44.03 ± 6.30	0.281
Average Time (s)	5.66 ± 0.23	5.90 ± 0.26	0.004 *	7.20 ± 0.79	7.34 ± 1.05	0.281
Peak Power (W)	656.14 ± 97.43	609.11 ± 82.15	0.159	231.39 ± 78.73	233.05 ± 85.44	0.876
Relative Power (W·kg^−1^)	8.91 ± 1.04	8.29 ± 0.98	0.148	4.15 ± 1.35	4.18 ± 1.47	0.876
Mean Power (W)	509.80 ± 69.36	454.44 ± 63.37	0.010 *	198.39 ± 71.68	193.19 ± 71.69	0.353
Relative Mean Power (W·kg^−1^)	6.93 ± 0.76	6.17 ± 0.74	0.009 *	3.55 ± 1.19	3.45 ± 1.19	0.337
FI (%)	35.99 ± 8.93	40.58 ± 11.92	0.143	25.01 ± 11.75	30.35 ± 14.30	0.326
Resting HR (beats·min^−1^)	111.00 ± 13.75	108.00 ± 11.37	0.318	118.00 ± 19.20	120.70 ± 24.06	0.501
Mean HR (beats·min^−1^)	166.63 ± 10.56	164.97 ± 8.53	0.600	168.00 ± 4.63	166.03 ± 8.34	0.352
HR 6th Set (beats·min^−1^)	177.50 ± 9.77	173.80 ± 8.04	0.174	175.70 ± 5.36	173.20 ± 8.22	0.164
RPE 6th Set	8.90 ± 1.10	9.30 ± 0.68	0.168	8.90 ± 0.88	8.50 ± 1.43	0.104

Values are means (M) ± standard deviation (SD). DC (dark chocolate); WC (white chocolate); Total Effort Time (seconds, s); Average Time (seconds, s); Peak Power (watts, W); Relative Power (watts per kilogram, W·kg^−1^); Mean power (watts, W); Relative mean power (watts per kilogram, W·kg^−1^); FI (Fatigue Index, percentage, %); resting HR (resting heart rate); mean HR (heart rate, beats per minute, beats·min^−1^); HR 6th set (heart rate, beats per minute, beats·min^−1^); RPE 6th set (rate of perceived exertion). * indicates significant difference between trials for gender in 2nd RAST (*p* < 0.05).

## Data Availability

The raw data supporting the conclusions of this article will be made available by the authors on request. The data are not publicly available due to privacy and ethical reasons.
